# The effect of low level laser on condylar growth during mandibular advancement in rabbits

**DOI:** 10.1186/1746-160X-8-4

**Published:** 2012-02-23

**Authors:** Mostafa Abtahi, Maryam Poosti, Nasrollah Saghravanian, Kamran Sadeghi, Hooman Shafaee

**Affiliations:** 1 Orthodontic Dept, School of Dentistry and Dental Research Center, Mashhad University of Medical Sciences, Mashhad, 91735 Iran; 2 Orthodontic Dept, School of Dentistry, Islamic Azad University, Tehran, Iran; 3 Oral and maxillofacial Pathology Dept, School of Dentistry, Mashhad University of Medical Sciences, Mashhad, 91735 Iran

**Keywords:** Low level laser, rabbit, bite jumping, condyle

## Abstract

**Introduction:**

It has been shown that Low Level Laser (LLL) has a positive effect on bone formation. The aim of this study was to evaluate the effect of low level laser on condylar growth during mandibular advancement in rabbits.

**Materials and methods:**

Continuous forward mandibular advancement was performed in fourteen male Albino rabbits with the mean age of 8 weeks and the mean weight of 1.5 ± 0.5 kg, with acrylic inclined planes. The rabbits were randomly assigned into two groups after 4 weeks. LLL (KLO3: wave length 630 nm) was irradiated at 3 points around the TMJ, through the skin in the first group. The exposure was performed for 3 minutes at each point (a total of 9 minutes) once a day for 3 weeks. The control group was not exposed to any irradiation. The rabbits in both groups were sacrificed after two months and the histological evaluation of TMJ was performed to compare fibrous tissue, cartilage, and new bone formation in condylar region in both groups. Disc displacement was also detected in both groups. Student's t-test, Exact Fisher and Chi square tests were used for the statistical analysis.

**Results:**

The formation of fibrous tissue was significantly lower, while bone formation was significantly greater in lased group as compared with control group. The thickness of cartilage did not differ significantly between two groups.

**Conclusion:**

Irradiation of LLL (KLO3) during mandibular advancement in rabbits, increases bone formation in condylar region, while neither increase in the cartilage thickness nor fibrous tissues was observed.

## Introduction

The Class II malocclusion has been called the most frequent skeletal problem in the orthodontic practice [1,2]. The solution can involve the use of functional or fixed orthodontic appliances, or both [3]. It has been claimed that the most frequent skeletal problem in Class II patients is mandibular retrognathia [4,5]. In the treatment of Class II malocclusion, capability to alter patients' facial growth is of particular interest, namely by means of functional appliances [6,7]. The findings from animal and human studies have been accepted as evidence that functional appliances can stimulate condylar [8-10] or mandibular growth,[11,12] and are able to make changes in the underlying skeletal pattern of the patient. Therefore the success of Class II treatment with mandibular deficiency depends on the ability of functional appliances to encourage condylar growth.

Quantitative histological studies have clarified the time-dependent nature of the adaptive response, indicating that the initial large changes in cartilaginous proliferation are progressively diminished when restoration of functional equilibrium is obtained [13].

The development of technologies capable of accentuating the growth potential of mandibular cartilage could allow our profession to predictably accelerate the growth phenomena of this tissue. One stimulus capable of improving this process is the application of low-intensity pulsed ultrasound [14,15].

Recently, low-level laser was used to enhance bone healing after fracture [16,17], after mandibular distraction osteogenesis,[18,19] and also for condylar growth stimulation [20]. The results suggest that Low level laser therapy(LLLT) had a positive effect on the percentage of newly formed bone. Better-quality bone sites may allow early healing, thus shortening total treatment time.

Considering the positive effects of LLLT on bone regeneration and the common tendency of shortening treatment period in orthodontics, the aim of the present study was to evaluate the effect of low level laser on condylar growth during mandibular advancement in rabbits. Our hypothesis was that LLLT could increase bone formation during mandibular advancement.

## Materials and methods

**Figure 1:**
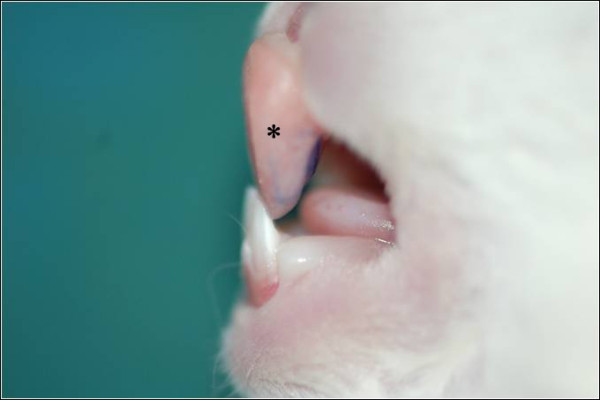
**bite jumper appliance**.

This study was approved by ethical committee of Mashhad University of Medical sciences. (Code: 88349). Fourteen male white Albino rabbits with the mean age of 8 weeks and the mean weight of 1.5 ± 0.5 kg were selected. All the animals had intact central incisors in the upper and lower arch. Under general anesthesia (intramascular injection of 1 ml Xylazine and Ketamin with 1:2 ratio) primary impressions were obtained from maxilla and after constructing special trays secondary impressions were taken and plaster models were made. Identical acrylic inclined planes were constructed for the anterior teeth of rabbits, to serve as functional appliances and create continuous forward mandibular advancement. These appliances were bonded to upper central incisors by self cure composite.(Figure 1)

Following bonding the bite jumper appliance, rabbits were randomly assigned into two groups of seven. In the first group 630 nm low level laser with 10 mw power and a probe diameter of 0.8 mm (KLO3 Mustang2000, Russia) possessing a continuous mode, was irradiated at 3 points around the TMJ, through the skin from the end of the 3^rd ^week after bite jumping [21]. Exposure was performed for 3 minutes [22] at each point (a total of 9 minutes) once a day for 3 weeks [23].

The control group was not exposed to any irradiation. After two months the rabbits in both groups were sacrificed by vital perfusion, the mandibles were dissected and fixed in formaldehyde 4%, decalcified in EDTA for 60 days and then embedded in paraffin. Serial sections from TMJ including condyle and glenoid fossa were cut sagitally with 4-5 μm diameter, and stained with hematoxylin and eosin (H&E) to determine the following criteria:

1- Maximum thickness of condylar fibrous tissue.(The number of fibroblasts and collagen bundles were determined in tissue;extensive seperation of fibroblasts by abunant collagen was considered as Fibrosis.)[24]

2- Minimum thickness of condylar fibrous tissue

3- Maximum thickness of condylar cartilage

4- Minimum thickness of condylar cartilage

5- Maximum thickness of condylar new bone

6- Minimum thickness of condylar new bone

7- Disc displacement

**Figure 2:**
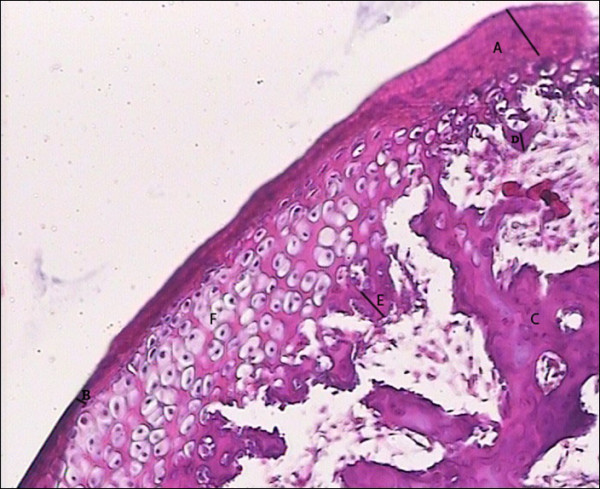
**A: Maximum thickness of condylar fibrous tissue B: Minimum thickness of condylar fibrous tissue C: old bone D: Minimum thickness of condylar new bone E: Maximum thickness of condylar new bone F: hyperthrophic chondrocytes**.

The sections were evaluated blindly under a light microscope (Leica BME) with ×100 magnification. The photograph of each section was taken and saved as a digital file, and then analyzed by Adobe Photoshop CS2 software (Adobe System Incorporated, USA). (Figure 2). The bone interconnected to cartilage considered as new bone. The power calculation for different variables to confirm the reliability of the study was performed.

After the normal distribution of data was confirmed by Kolmogrov-Smirnov test the data were analyzed by Student t-test, Exact Fisher and Chi square tests.

## Results

The power calculation for different variables included a follow: maximum condylar fibrous:0.99, minimum condylar fibrous: 0.70, maximum condylar cartilage: 0.35, minimum condylar cartilage: 0.12, maximum new bone: 1, minimum n new bone: 1. The power of our study for bone formation and condylar cartilage wa above 80% which was completely acceptable.

The results show that maximum and minimum fibrous tissue thickness in condylar region are statistically greater in control group as compared to lased group(p < 0.05), while maximum and minimum thickness of new condylar bone is statistically greater which shows more bone formation in the lased group (p < 0.01). There was no statistically significant difference found in the maximum and minimum of new cartilage formed in the condylar area(p > 0.05).(Table 1)

**Table 1:** Comparison of lased and control group condyles in different variables (mm)

Variable(mm)	Group	Mean	STD	Max	Min	P- value
Max thickness condylar fibrous tissue	L	1.40	0.46	0.90	2.10	0.00
	C	2.99	0.89	2.10	4.50	
	
Min thickness condylar fibrous tissue	L	0.59	0.25	0.25	1.10	0.014
	C	1.02	0.43	0.60	1.90	
	
Max thickness condylar cartilage	L	3.73	1.42	1.80	6.80	0.115
	C	4.83	1.41	2.80	7.10	
	
Min thickness condylar cartilage	L	1.41	0.91	0.75	4.10	0.413
	C	1.74	0.73	0.90	3.10	
	
Max thickness condylar new bone	L	19.29	1.63	16.20	21.60	0.00
	C	12.24	1.03	9.80	13.10	
	
Min thickness condylar new bone	L	5.04	0.97	3.50	6.50	0.00
	C	2.25	0.50	1.60	3.20	

## Discussion

In this study we clearly demonstrated the stimulatory effects of 630 nm low level KLO3 laser irradiation on bone formation in condylar region during mandibular advancement in rabbits. The data of this study suggests that newly formed bone was significantly increased by 3 weeks irradiation around TMJ during employing bite jumper appliance.

Rabie et al have shown that the best response of TMJ to mandibular advancement and the highest level of bone formation in the glenoid fossa was detected on day 21, so we started our laser irradiation on the third week [21].

Histological examination showed no pathological changes such as bone resorption in condylar area, and lower fibrous tissue formation in lased group indicates lower inflammation established in this group. Statistically significant greater amounts of bone were observed in the experimental group which strongly indicates that application of LLL accelerates the maturation of new bone tissue.

Miloro *et al *found that LLL accelerates the process of bone regeneration in the mandibles during the consolidation phase after distraction osteogenesis as compared with control animals [19].

Current theories suggest that transcription of certain nuclear proteins, such as a rhodopsin-kinase enzyme may be photosensitive at certain wavelengths and this may be responsible for the accelerated wound healing capabilities of the LLL [25].

The results of Stein's studies indicate that low-level laser therapy has a biostimulatory effect on human osteoblast-like cells [26] and it could promote proliferation and maturation of human osteoblasts in vitro [27]. Similar conclusions have been obtained by Dörtbudak about the effect of soft diode lasers on osteoblasts derived mesenchymal cells [28].

Liu believes that LLL may accelerate the process of fracture repair or increases the callus volume and bone mineral density, in the early stages of fracture healing [29].

Khadra et al claimed that the application of LLL with a GaAlAs diode laser device can promote bone healing and formation in skeletal defects [30].

Future studies are warranted with larger numbers of animals. Also, further research is needed to determine the precise cellular and biochemical effects of LLL treatment on both hard and soft tissues.

## Conclusion

Regardng the findings of this study LLL may prove efficacious in allowing a shorter period of functional therapy. Irradiation of LLL (KLO3) during mandibular advancement in rabbits, increases bone formation in condylar region, while no increase in the cartilage thickness or fibrous tissues was observed. This would provide great benefit to patients, allowing them to avoid the burdens of a prolonged treatment

## Competing interests

The authors declare that they have no competing interests.

## Authors' contributions

MA guided the practical parts of animal study and participated in study design, MP participated in the design of the study and prepared the manuscript, NS performed the histological study, KS accomplished the animal study, HS helped with the statistical analysis. All authors read and approved the final manuscript.
